# Preventing microbial adherence: An annotated selection of World Wide Web sites relevant to the topics in microbial biotechnology

**DOI:** 10.1111/1751-7915.12185

**Published:** 2014-10-29

**Authors:** Lawrence P Wackett

**Affiliations:** Department of Biochemistry, Molecular Biology and Biophysics, BioTechnology Institute, University of MinnesotaSt. Paul, MN, 55108, USA

## Silver surfaces to prevent adherence

### http://www.sciencedirect.com/science/article/pii/S0924857909000648

It is extremely important to develop surfaces that prevent microbial adherence in biomedical applications such as implanted medical devices and this article addresses that issue.

## Forces involved in bacterial adhesion

### http://mic.sgmjournals.org/content/154/10/3122.full.pdf

Bacterial adhesion to surfaces is very complex and there are a range of forces that govern this overall process. These issues are discussed and examined experimentally in this article.

## Nanosilver material preventing adhesion

### http://pubs.acs.org/doi/abs/10.1021/la303264k

Silver has long been known to inhibit bacteria and that element can now be used with advanced materials to prevent adhesion and subsequent biofilm build-up.

## Microbial adherence and fluid flow

### http://phys.org/news/2014-02-microbes-adhere-tube-walls.html

This article discussed recent studies on the behaviour of bacteria in turbulent flows and the unexpected finding that these conditions promote bacterial adhesion.

## Microbial adherence via flagella

### http://firstlook.pnas.org/flagellar-anchors-help-bacteria-adhere-to-surfaces/

Flagella are used to propel bacteria through water, but they can also serve to stick into the crevices of surfaces and thus attach and anchor bacteria of those surfaces, as discussed in this review.

## Surface nanostructure to control bacterial adherence

### https://aizenberglab.seas.harvard.edu/papers/Nanotechnology2011.pdf

This paper addresses the effect of surface structural patterning on microbial adhesion and the forces involved in those interactions.

## Microbial life on surfaces

### http://www.mrs.org/s11-abstract-kk/

This symposium website contains the participants and abstracts for a meeting discussing the interaction between materials and bacteria adherence to surfaces.

## Bacterial adherence studied by atomic force microscopy

### https://www.wpi.edu/Pubs/ETD/Available/etd-082206-162049/unrestricted/Arzu_Atabek_MSthesis.pdf

This thesis describes studies on the use of atomic force microscopy to study bacterial adherence.

## Chemistry of biofilm prevention

### http://en.wikipedia.org/wiki/Chemistry_of_biofilm_prevention

This site provides a general overview of bacterial biofilm formation and means to prevent it. References are provided.

## Physicochemical regulation of biofilm formation

### https://www.biochem.wisc.edu/faculty/weibel/lab/publications/pub_pdfs/2011_pubs/40_Renner_Weibel.pdf

This review article describes the stages of biofilm formation, going through initial stages of adherence to surfaces.

